# Efficacy of Concomitant Therapy with Fluoride and Chlorhexidine Varnish on Remineralization of Incipient Lesions in Young Children

**DOI:** 10.5005/jp-journals-10005-1381

**Published:** 2016-12-05

**Authors:** Sarika Naidu, Shobha Tandon, Rashmi Nayak, P Venkat Ratnanag, Deepesh Prajapati, Namitha Kamath

**Affiliations:** 1 Assistant Professor, Department of Pedodontics, SB Patil Dental College and Hospital, Bidar, Karnataka, India; 2 Professor, Faculty of Dentistry, University Technology MARA, Sungai Buloh Campus, Selangor, Malaysia; 3 Professor and Head, Department of Pedodontics, Manipal College of Dental Sciences, Manipal, Karnataka, India; 4 Reader, Department of Prosthodontics, SB Patil Dental College and Hospital, Bidar, Karnataka, India; 5 Assistant Professor, Department of Pedodontics and Preventive Dentistry, NIMS Dental College, Jaipur, Rajasthan, India; 6 Senior Lecturer, Department of Pedodontics, AB Shetty Memorial Institute of Dental Sciences, Mangaluru, Karnataka, India

**Keywords:** Chlorhexidine, Fluoride, Incipient lesions, Varnishes.

## Abstract

**Aim:**

To assess the effect of combined use of chlorhexidine and fluoride varnish on the remineralization of incipient carious lesions in young children.

**Materials and methods:**

Twenty caries-active children (80 lesions) were randomly divided into four groups and subjected to initial examination. Caries status was assessed visually and with the aid of DIAGNOdent. Baseline enamel biopsies were obtained. Subjects of groups I and II received fluoride and chlorhexidine varnish respectively. Group III received both fluoride and chlorhexidine varnish alternatively, for a period of 4 weeks. Group IV served as the control. At 3-month follow-up, the incipient lesions were assessed again with DIAGNOdent and enamel biopsy.

**Results:**

Increased calcium, phosphate, and fluoride levels were noticed in groups I, II, III compared to group IV, at the 3-month follow-up (p < 0.001).

**Conclusion:**

The combined therapy with fluoride and chlorhex-idine varnish may be considered an alternative therapy for early reversal of incipient lesions.

**How to cite this article:**

Naidu S, Tandon S, Nayak R, Ratnanag PV, Prajapati D, Kamath N. Efficacy of Concomitant Therapy with Fluoride and Chlorhexidine Varnish on Remineralization of Incipient Lesions in Young Children. Int J Clin Pediatr Dent 2016;9(4):296-302.

## INTRODUCTION

Fluoride is the most prominent drug used as an auxiliary preventive measure, which exerts its action by enamel remineralization. Modern concepts of the mechanism of action of fluoride recommend daily fluoride supplies to establish and maintain a significant concentration in saliva and plaque fluids, thus preventing and controlling enamel dissolution. However, isolated use of fluoride has proved to be insufficient to prevent progressive mineral loss and consequent lesion formation in children at a high risk for caries development.

Chlorhexidine is a cationic agent, and its hydro-phobic and hydrophilic properties are responsible for its efficacy. Chlorhexidine binds readily to negatively charged bacterial cell walls and can thereby disrupt the membrane integrity. In high concentrations, chlorhexi-dine is bactericidal and acts as a detergent by damaging cell membranes. It also has the ability to be retained on oral surfaces and is gradually released into oral fluids over many hours. Chlorhexidine can effectively reduce *Streptococcus mutans* levels in the plaque biofilm. Chlorhexidine controls the plaque formation and, at the same time, reduces its acidogenicity, thereby increasing the possibility of remineralization of the white spots.^1^

It has been proposed that combinations of two or more anticaries agents may have an additive protective effect if each agent has a different site of action. Acid production is an important factor in the pathogenesis of dental caries. As fluoride and chlorhexidine inhibit this process at different steps, these two agents exert additive inhibitory effects. Based on these principles, the fluoride-chlorhexidine association would prove to be quite beneficial, with chlorhexidine-reducing plaque acid formation for several hours and preventing the decrease in pH and fluorides aiding in the remineralization process.^2^

The present study was designed with aims to assess the effect of the concomitant use of fluoride and chlorhexi-dine varnish on the incipient lesion remineralization in young children and to assess the efficacy of DIAGNOdent in monitoring the remineralization of incipient lesions following preventive therapy.

**Flow Chart 1:**
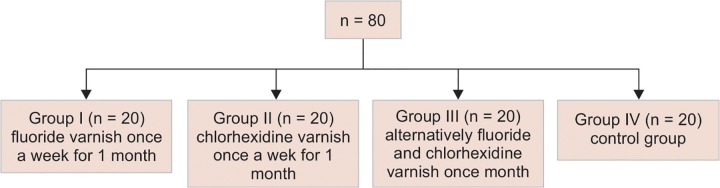
Distribution of the sample size

## MATERIALS AND METHODS

The study was based on demonstrating a 5% difference of remineralization in between the groups, which was thought to be significant. To give 80% power and a 5% significance level, using a two-sided analysis of variance (ANOVA) test, a sample size of 15 in each group was calculated as sufficient to detect the difference between the groups.

To generate minimum of 15 samples per group, a convenient sample of 80 primary teeth (20 in each group) with a white spot on each tooth were selected according to the inclusion criteria. Protocol approval was obtained from institutional Ethics Committee. The parents of participating children were clearly explained the purpose of the study, and informed consent from the principal of the school as well as the parents of the students participating in the study was obtained.

The inclusion criteria for the study population were healthy children of age group 3 to 6 years with incipient lesions, whose parental consent was obtained; similar socioeconomic status; dietary pattern; and similar oral hygiene practice, i.e., brushing their teeth two times a day. However, in children with long-term systemic illness, previous history of treatment of dental caries was excluded.

Eighty teeth with incipient lesions of primary dentition were divided into four groups, as shown in the Flow Chart 1.

A clinical examination of all the subjects participating in the study was carried out using a mouth mirror, perio-dontal probe and airway syringe by a single examiner. Dental caries status was recorded according to dental staining index.^3^ Only white spot lesions of smooth surfaces of primary teeth were considered in the study.

**Fig. 1:**
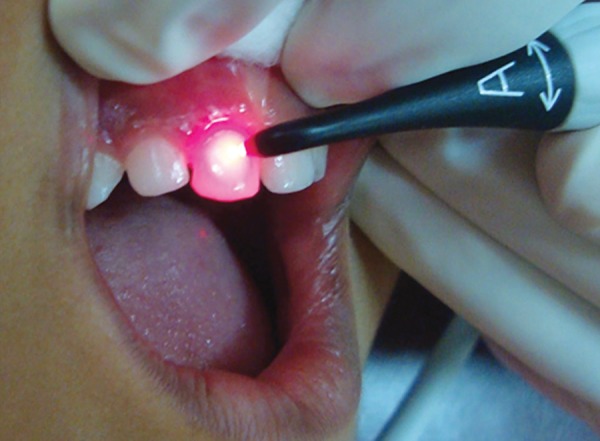
Examination with DIAGNOdent

Once the teeth were prophylactically cleaned and rinsed thoroughly, surfaces of test sites were examined using DIAGNOdent according to manufacturer’s instructions (Fig. 1). Probe ‘B’ was used under cotton roll isolation and after air drying with an air syringe.

**Table 1:** **Table 1:** Manufacturer’s cutoff points for DIAGNOdent used in this study

*Score*		*Fluoroscence values*		*Clinical criteria*	
D0		0-5		No demineralization	
D1		6-14		Outer enamel demineralization	
D2		15-20		Inner enamel demineralization	
D3		21-99		Dentin demineralization	

The instrument was calibrated using ceramic mounting that was provided by manufacturer. Probe ‘B’ was placed perpendicular to the test site and rotated along the fissure to completely scan the area. Base line reading for each tooth was taken by placing the probe on sound tooth surface. Three measurements were taken, and mean of them was considered as final base line value. Both moment and peak values were recorded. Three measurements were performed for each tooth, and mean of them was taken. This value was then subtracted from base line value to attain the final value. Then scoring was given according to the cutoff limits given by manufacturer (Table 1).

**Fig. 2:**
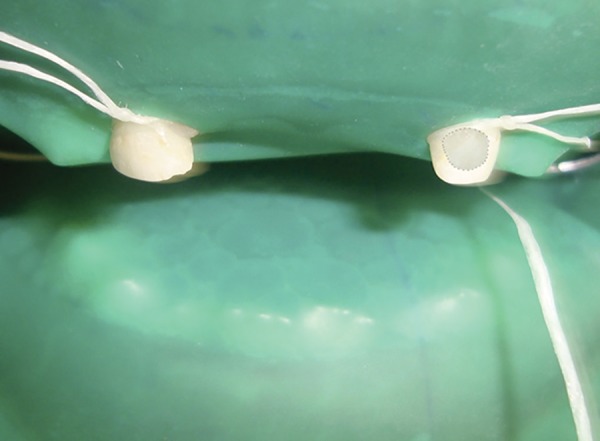
Procedure for enamel biopsy

Following examination with DIAGNOdent, enamel biopsy was taken as recommended by Vogel et al.^4^ The tooth was isolated with the help of rubber dam to eliminate any chances of saliva contamination (Fig. 2). A 4-mm-diameter nonfluoride-containing filter paper circle was wetted with 0.5 mol/L perchloric acid and immediately placed on the test site of the tooth for 15 seconds using a timer. This filter paper was then transferred to sterile container which had 20 mL of pipetted double-distilled water. The enamel biopsy samples were stored in sterile bottles containing 20 mL of double-distilled water and were refrigerated at a temperature of 0° Celsius until it was transferred to the laboratory.

**Fig. 3:**
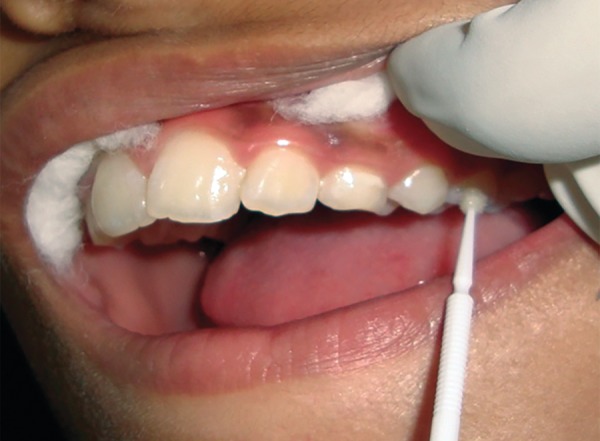
Varnish application

After the baseline assessment, group I received fluoride varnish once a week for 1 month (Fig. 3), Group II received chlorhexidine varnish once a week for 1 month, group III received alternate week application of fluoride and chlorhexidine varnish for 1 month, and group IV served as negative control group.

Application of fluoride and chlorhexidine varnish was done in following steps: Tooth surfaces were cleaned thoroughly, isolated with cotton rolls, and dried with air syringe. Three drops were pressed out into dappen dish. A thin coat of varnish was applied by means of a vivadent applicator. Proximal areas were coated with dental floss. Varnish was allowed to dry and cotton rolls removed after 30 seconds. Patients were instructed not to rinse the mouth and to avoid eating for the next 2 hours. Children were advised to take soft diet and not brush for the rest of the day.

Stored enamel samples were transported to chemical engineering department for mineral analysis within 15 days.

Calcium levels were estimated using atomic absorption spectrophotometer. The atomic absorption spectrophotom-eter uses the absorption of light to measure the concentration of gas phase atoms. From the liquid samples (20 mL), the analyte atoms or ions were first vaporized in a flame, to facilitate the absorption of ultraviolet or visible light from the light source and make transition to higher electronic energy levels. The analyte concentration was determined from the amount of absorption by the atoms/ions. Concentration measurements were determined from the working curve obtained after calibrating the instruments with the standards of known concentration.

Phosphate and fluoride levels were estimated using ICS-90 Ion Chromatography System (ICS-90). The chro-matography software (for the ICS-90, this is Chrome-leon^®^) analyzes the data by comparing the sample peaks in a chromatogram to those produced from a standard solution. The software identifies the ions based on retention time, and it quantifies each analyte by integrating the peak area or peak height.

At 3-month follow-up, the examination with DIAG-NOdent and estimation of mineral content using enamel biopsy for incipient lesions were repeated. On the same visit, the children were provided with the required treatment.

The collected data were subjected to statistical analysis. For intragroup comparison of the paired sample, t test was applied, and for the intergroup comparison, one-way ANOVA, followed by Tukey’s test, was applied. All the tests were carried out using the Statistical Package for the Social Sciences (SPSS) package in the computer. The parameters, which were evaluated, are calcium, phosphate, and fluoride levels of enamel biopsy and DIAGNOdent values of the incipient lesion.

## RESULTS

The mean age of the subjects in group I (fluoride varnish group) was 5.70 ± 1.30, group II (chlorhexidine varnish group) was 4.65 ± 0.67, group III (fluoride and chlorhexidine group) was 5.0 ± 0.56, and group IV (control group) was 4.65 ± 0.49. The difference was not statistically significant between the groups.

**Graph 1:**
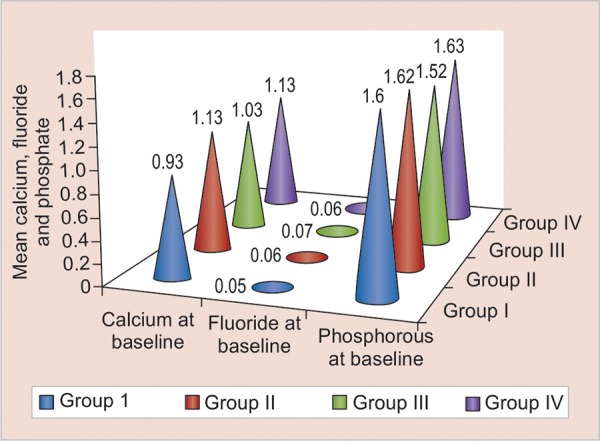
Mean calcium, fluoride, and phosphate levels at baseline

**Graph 2:**
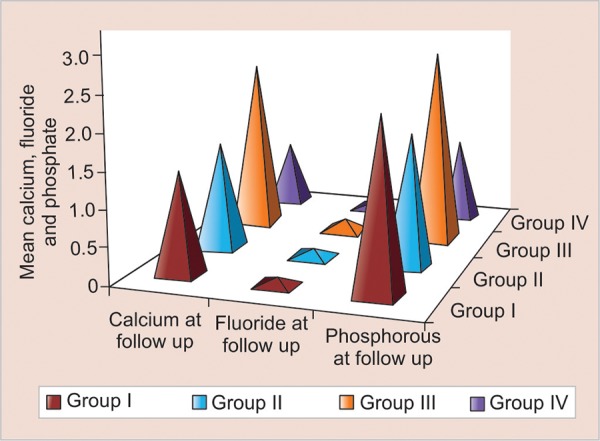
Mean calcium, fluoride, and phosphate levels at follow-up

**Graph 3:**
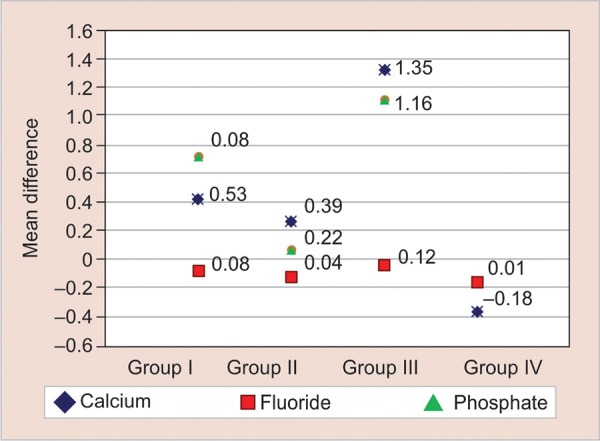
Mean differences of calcium, phosphate, and fluoride in all the groups

Mean levels at baseline and at follow-up of calcium, fluoride, and phosphate have been shown in Graphs 1 and 2, with mean differences in Graph 3.

**Graph 4:**
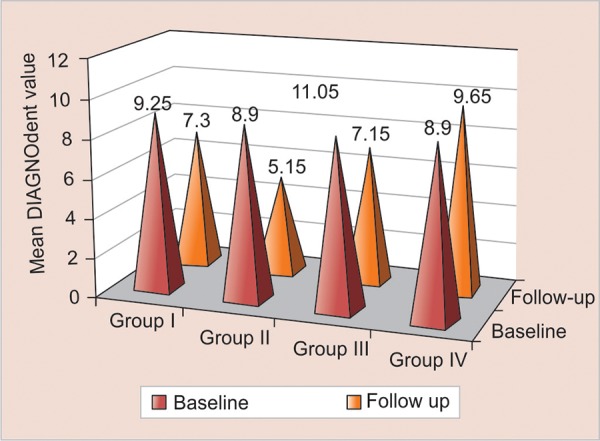
Mean DIAGNOdent values at baseline and follow-up among the study groups

Table 2 shows the clinical findings of the white spot lesions recorded at baseline and at the 3-month follow-up.

DIAGNOdent values reduced in all the three groups as compared to the control group (Graph 4).

Overall, results of the present study showed that the fluoride, chlorhexidine, and combination of varnish groups demonstrated increased calcium, phosphate, and fluoride levels as compared to the negative control group, at 3-month follow-up.

**Table 2:** **Table 2:** Percentage reduction of white spot lesions after preventive intervention

		*Baseline*				*Follow-up*					
*Groups*		*No. of* * teeth*		*No. of* * white spot* * lesions*		*No. of* * teeth*		*No. of* * white spot* * lesions*		*Reduction* *of white spot* * lesions (in %)*	
I		20		20		20		12		40	
II		20		20		20		14		30	
III		20		20		20		9		55	
IV		20		20		20		20		0	

## DISCUSSION

In this study, children with similar socioeconomic backgrounds and having almost similar dietary practices and oral hygiene practices were chosen so as to minimize the effect of any other etiologic factors in the progression and regression of incipient lesions. Dietary practices were assessed using the method given by Johansson et al.^5^ According to this, all the subjects in the study had similar dietary practices.

DIAGNOdent was used to monitor the remineraliza-tion of incipient lesions. DIAGNOdent is a dependable tool for monitoring the progression and regression of incipient lesions, following preventive therapy.^6^ Therefore, in our study, to avoid any discrepancies, the results of lesion progression/regression using DIAGNOdent have been supplemented with acid enamel biopsies.

Post oral prophylaxis enamel biopsies were carried out for the analysis of the mineral content in the surface layer of the incipient lesion, as described in previous studies.^4,7^ Perchloric acid has been used to quantify the mineral content in the superficial layer of the tooth surface. The whole procedure was done under rubber dam isolation in order to prevent acid exposure to the soft tissues.

If the varnish on the tooth surface is retained for a prolonged period, increased fluoride concentrations produce deposits of calcium fluoride-like material. Fluoride from this calcium fluoride material, which is deposited in the porosities and cariogenic sites in enamel, can gradually diffuse into overlying dental plaque or underlying enamel. Cariogenic sites specifically adsorb fluoride and subsequently release it for a certain period of time. Fluoride release can prevent demineralization and promote remineralization of early initial caries lesions.^8^ Varnishes are also relatively easy to apply and well tolerated, especially by children, hence in this study, varnishes have been selected as the means to deliver the preventive therapy.

A previous study has shown that fluoride varnish enhances remineralization of active white spots because the quantities of calcium and phosphate lost by dental structure can be replaced in the form of fluorapatite in the enamel.^9^

In the present study, in group I, where fluoride varnish was applied, it was found that there was a highly significant increase in the calcium, phosphate, and fluoride scores from baseline to the end of 3 months (p < 0.001) (Graphs 1-3). These findings were in accordance with the previous studies conducted, which reported significant remineralization of early enamel lesions with 5% sodium fluoride varnish.^10,11^

A 51% reversal of decalcified tooth structure and a 35 to 21% reduction in enamel demineralization with fluoride varnish has been observed in another study.^12^ Another report confirmed that fluoride varnish application was effective in reversing and arresting active enamel lesions and, therefore, reduced the need for restorative intervention.^10^ Similarly, in the present study, a 40, 30, and 55% reduction in the number of white spot lesions was observed in groups I, II, and III respectively at 3-month follow-up. This can be attributed to the remin-eralization caused by the varnish used.

In the present study, four weekly applications of varnish was done initially for 1 month only to confirm the benefits of repeated fluoride application for a short period of time, and the effect at 3-month follow-up was observed. Similar methodology has been used in previous studies as well following it for 6 months.^13,14^

Long-term suppression of *S. mutans* in dental plaque and saliva by chlorhexidine varnishes has been reported earlier.^15^ Chlorhexidine varnish has been designed to reduce plaque accumulation on caries-susceptible sites, especially on exposed root surfaces.^16^ The earliest chlorhexidine varnish study that was done used chlorhexidine, with the trade name of Cervitec, in an attempt to reduce fissure caries in permanent molars.^17^ The results of the study showed a significant reduction in caries, which was correlated to the decreased level of plaque *S. mutans* at the occlusal sites. Another clinical evaluation conducted also arrived at the same conclusion. In view of these findings, chlorhexidine varnish was selected as a preventive agent against incipient lesions for this study.^18^

Findings of group II suggest that chlorhexidine varnish may be effective in enhancing remineralization process of incipient lesions since there was significant increase in the calcium, phosphate, and fluoride levels. Remineralization of incipient lesions after repeated applications of chlorhexidine varnish has already been proved before.^2,6^

Chlorhexidine reduces the microorganisms and, as a result of this action, a favorable pH is maintained.^2^ The findings of the present study show that although chlorhexidine caused remineralization of the lesions, it was to a lesser extent than that caused by fluoride varnish (Table 2; Graphs 1-3). This difference in the remineraliza-tion effect may be due to the inability of chlorhexidine varnish to supplement the required mineral content.

In group III, both fluoride and chlorhexidine were applied once in every week, alternatively for a period of one month. Results showed highly significant increases in calcium, fluoride, and phosphate levels (p < 0.001) at the end of 3 months compared to the base line levels (Graphs 1-3). This indicates a beneficial action taking place with the simultaneous use of both fluoride and chlorhexidine.

A combination of both the varnishes was selected in this study as it has shown that the isolated use of fluoride proved to be insufficient to prevent progressive mineral loss and consequent lesion formation in children at high risk for caries development.^19,20^ The results also showed that the combination of chlorhexidine and fluoride was more effective in remineralizing the white spots than the application of fluoride or chlorhexidine alone (Table 2; Graphs 1-3). Previous studies also attained similar results in irradiated patients who are considered to be of high risk for caries.^21,22^

The concomitant use of varnish with fluoride and chlorhexidine seems to create a favorable environment for remineralizing incipient lesions. Chlorhexidine, with its antiplaque effect and its specific action on *Streptococcus mutans,* would be responsible for the production of decreased levels of acid in the proximity of the active white spot lesion.^23^ With a smaller drop in plaque pH and the availability of fluoride ions released by the fluoridated varnish, there would be mineral replacement in the enamel in the form of fluorapatite.^24^

A study compared the efficacy of a chlorhexidine-thymol containing varnish with a chlorhexidine-thymol-fluoride containing varnish in decreasing interdental levels of *S. mutans.* It suggested that the addition of fluoride to an antibacterial varnish might improve the long-term efficacy of the varnish in diminishing the car-iogenic microbial challenge.^25^

A study in primary teeth showed that the combination chlorhexidine and fluoride in varnishes was more effective in reducing the plaque index and enhancing white spot remineralization after 3 months of observation, as compared to the isolated use of varnishes.^2^

In group IV, it was found that there was no significant increase in the calcium and fluoride levels from the baseline to the end of 3 months, as p value was 0.213 and 0.539 respectively. There was, however, a significant increase in phosphate scores at the end of 3 months showing a p value of 0.006 (Table 2; Graphs 1-3).

The present study has shown that professional cleaning (group IV) is not an effective means for remineralization of incipient lesions. In a study, following professional cleaning and application of fluoride varnish, there was significant increase in the oral hygiene scores in the subjects at subsequent visits. These authors stated that the remineralization of white spot lesions was due to the increase of calcium fluoride oral reservoirs provided by the fluoride varnish applications, the subsequent solubilization of these reservoirs, and the release of fluoride ions at low pH.^12^

Thus it can be concluded that through mechanical removal of plaque, the tooth surface was exposed to naturally occurring process of remineralization, but it seemed to be too slow to produce any significant increase in the mineral content of the incipient lesion.

Groups I, II, and III showed a significant decline in DIAGNOdent values at the end of 3 months when compared to baseline DIAGNOdent values (p < 0.008, < 0.001, and < 0.001 respectively) (Graph 4). This shows that the preventive therapy with the use of fluoride and chlorhexi-dine varnishes that were used have dramatically helped in remineralization of the incipient lesions.

As DIAGNOdent allows early detection of caries, and this helps to introduce noninvasive preventive measures to control the development of caries,^26^ the use of DIAGNOdent may also be suitable for longitudinal quantification of caries sites and to monitor the effects of an intensive prophylaxis regime.^27^ The present study has shown that the DIAGNOdent system is a sensitive clinical method which may be suitable for longitudinal quantification of smooth surface incipient lesions. These results were in accordance with another study, in which it was found that the regression of incipient carious lesions in smooth surfaces treated by topical fluoride applications can be monitored with laser fluorescence.^28^ Furthermore, it has been proved that significant reduction in DIAGNOdent readings occur over a time in molars treated with antibacterial varnish, suggesting that it is an alternative way of monitoring the outcome of caries-preventive measures.^29^

## CONCLUSION

The following conclusions were derived from the study:

- The concomitant use of fluoride varnish and chlorhex-idine varnish was found to be more effective in remineralizing incipient lesions when compared to the application of each varnish individually.
- The use of DIAGNOdent for assessing the reminer-alization of incipient lesions, following preventive therapy, is a reliable noninvasive monitoring tool.

However, further investigations are required to firmly establish the accuracy and reliability of DIAGNOdent in supervising the remineralization efficacy of various preventive methods. This can be done by studies carried out using a larger sample size and more frequent follow-ups extending for a longer duration.
